# Insights into the evolution of bacterial flagellar motors from high-throughput *in situ* electron cryotomography and subtomogram averaging

**DOI:** 10.1107/S2059798318007945

**Published:** 2018-06-01

**Authors:** Florian M. Rossmann, Morgan Beeby

**Affiliations:** a Department of Life Sciences, Imperial College London, London SW7 2AZ, England

**Keywords:** low-abundance imaging, electron cryotomography, subtomogram averaging, bacterial flagellar motors, molecular evolution

## Abstract

*In situ* structural information on molecular machines can be invaluable in understanding their assembly, mechanism and evolution. Here, the use of electron cryotomography (ECT) to obtain significant insights into how an archetypal molecular machine, the bacterial flagellar motor, functions and how it has evolved is described. Over the last decade, studies using a high-throughput, medium-resolution ECT approach combined with genetics, phylogenetic reconstruction and phenotypic analysis have revealed surprising structural diversity in flagellar motors. Variations in the size and the number of torque-generating proteins in the motor visualized for the first time using ECT has shown that these variations have enabled bacteria to adapt their swimming torque to the environment. Much of the structural diversity can be explained in terms of scaffold structures that facilitate the incorporation of additional motor proteins, and more recent studies have begun to infer evolutionary pathways to higher torque-producing motors. This review seeks to highlight how the emerging power of ECT has enabled the inference of ancestral states from various bacterial species towards understanding how, and ‘why’, flagellar motors have evolved from an ancestral motor to a diversity of variants with adapted or modified functions.

## Abstract 2

The use of high-throughput *in situ* electron cryotomography and subtomogram averaging to study the the architecture and diversity of the bacterial flagellar motor is reviewed. Together with phenotypic analysis, this information can be used to better understand the evolution of molecular machines.

## 1. Introduction   

Understanding how molecular machines evolve is important for reasons ranging from antibiotic design to synthetic biology. The bacterial flagellar motor is an ideal model system for probing the principles of molecular evolution. The flagellar motor powers the rotation of bacterial flagella, which are helical proteinaceous filaments extending from the bacterial cell body that act as propellers for bacterial propulsion through liquid medium or swarming across surfaces (Jarrell & McBride, 2008 ▸). The flagellar motor is widespread, enabling cross-species comparison, and well characterized in terms of its components and function (Ohnishi *et al.*, 1997 ▸; Imada *et al.*, 2016 ▸; Khan *et al.*, 1992 ▸; Thomas *et al.*, 2006 ▸), opening the way for deeper questions on molecular evolution.

Structure determination of molecular machines such as the flagellar motor is crucial to fully understand how they have evolved. Electron cryomicroscopy (cryo-EM) has become the technique of choice to gain structural insights into such large macromolecular complexes. While single-particle analysis cryo-EM (SPA) involves the purification of protein complexes for imaging (Bai *et al.*, 2015 ▸; Passmore & Russo, 2016 ▸), a related technique called electron cryotomography (ECT) together with subtomogram averaging (STA) can be used to obtain three-dimensional structures of molecular machines *in situ* without requiring a large number of particles (Ferreira *et al.*, 2018 ▸; Oikonomou & Jensen, 2017 ▸; Briggs, 2013 ▸). ECT involves flash-freezing intact cells and imaging them over a range of angles, while maintaining them in a frozen state, in an electron microscope. The resultant data set can be used to calculate a three-dimensional reconstruction of the sample, or tomogram. Subsequently, identical particles from multiple tomograms can be extracted, computationally aligned and averaged, yielding a three-dimensional reconstruction of the particle of interest with a higher signal-to-noise ratio, a technique that is particularly valuable for many membrane-associated machines that are difficult to purify intact.

Here, we review how ECT has contributed to our understanding of bacterial flagellar evolution by describing ECT, how the ECT workflow has been optimized to image these relatively low-abundance particles, and the resulting insights into motor diversity and evolution. We start with an overview of ECT and STA, outline how sample preparation, data collection and data analysis have been optimized for the problem, and describe how ECT has contributed to the understanding of flagellar motor diversity and evolution.

## 2. Electron cryotomography and subtomogram averaging   

**Figure 1:**
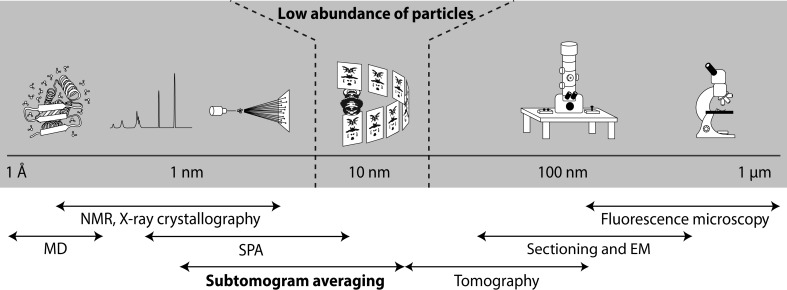
Schematic depicting electron cryotomography and subtomogram averaging of rare particles in the structural biology continuum. ECT bridges the gap between high-resolution structural biology techniques requiring the presence of a high abundance of particles and the low-resolution techniques used in cell biology. (EM, electron microscopy; MD, molecular dynamics; NMR, nuclear magnetic resonance spectroscopy; SPA, single-particle analysis.)

ECT is a technique that spans scales from structural biology to cell biology (Fig. 1 ▸). Cryo-EM involves the vitrification of a specimen by rapid freezing, preventing the formation of damaging ice crystals. This results in a sample suspended in a thin layer of vitreous ice that immobilizes the sample in a hydrated, close-to-native state that is stable for imaging in an electron cryo-microscope (Dubochet, 2012 ▸). Unlike conventional transmission electron microscopy, which requires chemical fixation and staining of the specimen, contrast in cryo-EM is derived from induced phase contrast of biological material in the microscope. Two major approaches are applied to obtain the molecular structure of large proteins or protein complexes using cryo-EM: SPA and ECT. SPA involves imaging many thousands of identical purified particles that are randomly oriented in vitreous ice. Given sufficient different orientations, it is possible to reconstruct a three-dimensional structure from these many two-dimensional images, in the process averaging out noise. Recent improvements in the developments of direct electron detectors have enabled full realization of this potential: the so-called ‘resolution revolution’ (Grigorieff, 2013 ▸; Ruskin *et al.*, 2013 ▸; Kühlbrandt, 2014 ▸). SPA can now provide very high resolution structures of protein complexes (2–4 Å).

ECT, unlike SPA, offers the ability to determine macromolecular structures such as the flagellar motor in their native crowded cellular context. Although SPA is capable of high-resolution structure determination, it requires purification of the sample outside the cellular environment. ECT, on the other hand, enables the study of large molecular machines *in vivo*. In ECT, the sample is tilted over a range of angles in the electron microscope and images are acquired at each step; the resulting tilt series is subsequently reconstructed into a three-dimensional tomogram of the specimen. Although the collection of a single tomogram typically takes 10–60 min, meaning that data acquisition is considerably slower than that in SPA, insights can be obtained into the three-dimensional architecture of unique specimens such as intact cells, enabling the extrapolation of details of individual components of the specimen that would be lost from a single two-dimensional projection image, and distinguishing the cellular context from the specimen of interest.

Individual tomograms have high levels of noise, necessitating strategies to extract the signal. The sensitivity of the specimen to ionizing electron radiation necessitates restriction of the electron dose during imaging, leading to individual tomograms with noise levels that obscure high-resolution information. Noise can be reduced by averaging the information from many identical structures across multiple tomograms. The structures of interest, referred to as particles, can be aligned using salient low-resolution features that are readily identifiable even under high-noise conditions and averaged, ‘washing out’ noise to significantly improve the signal-to-noise ratio. At best, such an approach enables access to high-resolution signal at ‘near-atomic’ resolution (<4 Å; Turoňová *et al.*, 2017 ▸), although resolutions are more typically in the nanometre range (Hu *et al.*, 2017 ▸; Beeby *et al.*, 2016 ▸). This process is called subtomogram averaging.

The use of ECT and STA has a number of advantages. Firstly, *in situ* imaging avoids the need for the development of bespoke purification protocols for variants of a specimen from mutants or different organisms. In addition, vitrification of a living cell provides a snapshot of a fully functional cell and its constituent machinery. Furthermore, tomograms provide additional information about the cell-biological context of a specimen, such as transient interactions with membranes, peptidoglycan or transiently interacting partners. ECT can also visualize fragile assembly intermediates and the heterogeneity of molecular machines *in situ* that would not be possible to purify, enabling routine genetic manipulation to perturb structure and function.

**Figure 2:**
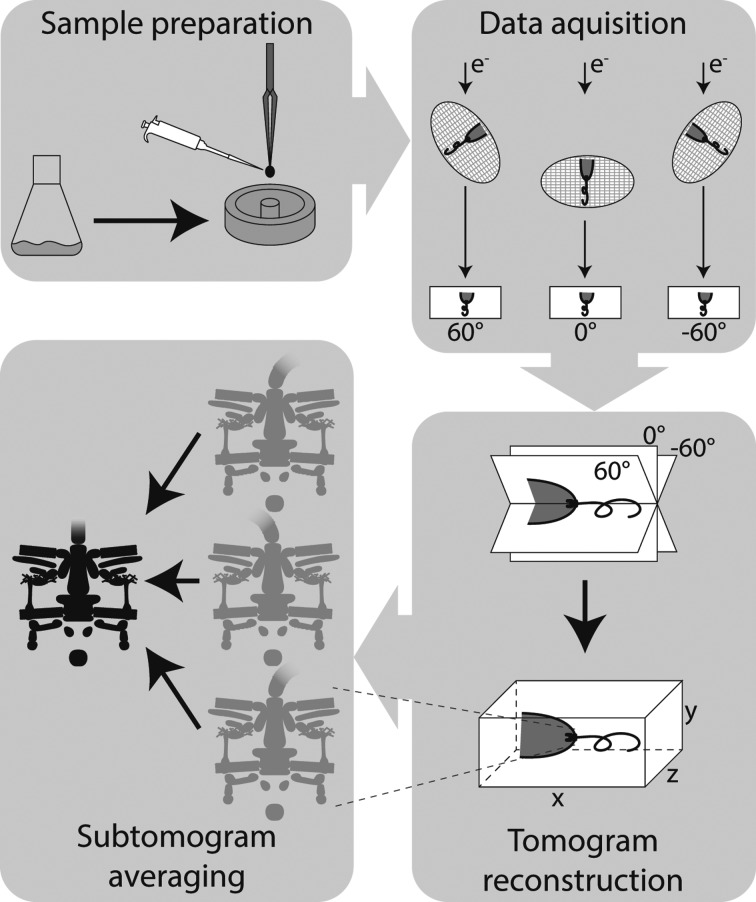
Illustration of the general workflow of ECT and STA to study bacterial flagellar motors. Schematic showing the different steps including sample preparation, data acquisition, tomogram reconstruction and subtomogram averaging. Samples are plunge-frozen in liquid cryogen, transferred to the microscope for the acquisition of images of cells over a range of angles and computationally reconstructed to form a tomogram; finally, identical structures from different cells are superimposed and averaged to yield a subtomogram average.

ECT with STA has become a powerful technique for determining the structures of bacterial flagellar motors, and their variants and mutants, to ‘macromolecular’ resolution (1–5 nm). Although sufficient particles in extremely thin samples can achieve resolutions below 4 Å (Turoňová *et al.*, 2017 ▸), the limited abundance of flagellar motors and the thickness of the cell currently makes it difficult to improve upon nanometre resolutions. To achieve this, optimization of sample preparation, automation of data acquisition, automation of tomogram reconstruction and a streamlined subtomogram averaging pipeline have been developed (Fig. 2 ▸).

### 2.1. Sample selection and preparation   

In bacterial tomography projects, it is essential to select a model system suitable for the collection of sufficient high-quality data to produce a sub­tomogram average of sufficient resolution. This requires the initial screening of possible candidate species, and not all interesting model organisms are suitable for further analysis. As vitrification of bacterial cells on electron-microscopy grids requires cultures with high cell densities, it must be possible to grow or concentrate the bacteria to a high cell density. Furthermore, the species must assemble sufficient functional flagella. Overexpression of transcriptional regulators or certain flagellar proteins or the deletion of negative regulators have successfully been used to increase the number of particles per cell (Liu *et al.*, 2012 ▸; Zhu *et al.*, 2017 ▸). Purification or enrichment protocols can further increase the number of flagellated cells, such as density-centrifugation approaches for minicells, as described fully below.

Because noise in cryo-EM images is a product of the thickness of the sample and its vitreous envelope, careful selection of the model bacterium plays a role in reducing specimen and ice thickness. Thin bacteria, and bacteria with lower turgor pressure that have a tendency to flatten in the thin layer of vitreous ice, are therefore amenable to ECT (Beeby *et al.*, 2016 ▸), leading to considerably thinner specimens along the axis of the electron beam. Flagella positioned at bacterial poles are also preferable to lateral flagella owing to the fact that, when oriented correctly, pole thickness does not increase as much upon tilting. Alternatively, genetic manipulation of genes involved in the cell-division machinery can generate thin, flagellated minicells (Farley *et al.*, 2016 ▸; Liu *et al.*, 2012 ▸). Optimizing the growth medium can also produce thinner cells (Chien *et al.*, 2012 ▸) or reduce clumping (Calleja, 2017 ▸), leading to decreased ice thickness and making motors more accessible for imaging. Screening many candidate species under different conditions with conventional negative-stain TEM techniques allows the selection of optimally thin bacteria with many flagella.

After identifying an optimal strain, the next parameter to maximize is the number of targets on an individual electron-microscopy grid. This optimization allows the generation of grids with hundreds of targets for tilt-series acquisition, maximizing the time for data collection and minimizing the time required to change grids in the microscope. Prior to vitrification, the sample is applied onto an electron-microscopy grid: a copper or gold grid consisting of 40–50 µm squares covered with a thin support film of carbon or gold. Support films have micrometre-scale holes in them, in which bacterial cells accumulate, allowing direct imaging of the cells without additional scattering of the electron beam through the support film. A few microlitres of bacterial suspension are applied to the grid, and excess liquid is removed using blotting paper, resulting in bacteria suspended in holes in the grid in a thin film of liquid. Iterative optimization of the blotting parameters is necessary for each sample to develop a reliable protocol for the generation of grids with hundreds of tomography targets. Vitrification robots help to maintain reproducible conditions and allow quantitative and reproducible settings for the blotting parameters to be found. Specifically, adjustment of the blotting time and the force of blotting maximizes the area of vitreous ice that is usable for tilt-series acquisition on the grid. In order to subsequently reconstruct tomograms from tilt series, nanoscale gold fiducial markers are also usually added to the sample immediately prior to vitrification. Optimization of the gold fiducial preparation leads to a reproducible, even distribution of a large number of fiducials per tomogram. Ethane cooled by liquid nitrogen can be used for vitrification; alternatively, a mixture of ethane and propane can be used, avoiding the need to monitor and control the ethane temperature to avoid ethane freezing near liquid-nitrogen temperatures (Tivol *et al.*, 2008 ▸).

### 2.2. Optimization of data acquisition and processing   

Streamlined data-acquisition pipelines are critical to facilitate rapid and reliable targeting. The use of high-throughput data-acquisition software such as *Leginon* (Suloway *et al.*, 2009 ▸) or *UCSF Tomo* (Zheng *et al.*, 2009 ▸) follows a ‘low-dose’ philosophy in which low electron-dose images are acquired once and stored for subsequent targeting, circumventing the need to expose parts of the grid to the electron beam multiple times. Such an approach facilitates a streamlined targeting process in which a mosaic of low-magnification images can be collected of the entire grid, enabling the construction of a ‘grid atlas’ montage to provide an overview of the entire grid for the iterative targeting of higher magnification images of grid squares, grid holes and targets for tilt-series acquisition. Electron-microscope presets describing the complete configuration of the microscope at different magnifications can be developed for optimal targeting at each preset magnification. Such an approach ensures that only cells with suitably oriented flagella are targeted.

Image contrast in the electron microscope is less straightforward than in a visible-light microscope (Ferreira *et al.*, 2018 ▸). Biological samples in an electron microscope provide little amplitude contrast, and phase contrast is negligible when the sample is focused within the electron microscope. When the image is underfocused, however, phase contrast becomes appreciable, although contrast varies as a function of defocus and the resolution of features, as mathematically described by the so-called ‘contrast transfer function’ (CTF). In broad terms, higher defocus values provide higher signal at lower resolutions but reduced signal at higher resolutions; since the lower resolution features of particles are required for accurate alignment, data-collection settings must balance defocus to optimize the ability to align samples but also retain sufficient high-resolution data. Furthermore, while increased electron dose increases the signal-to-noise ratio of an image, the ionizing nature of electrons means that too high a dose leads to specimen damage and therefore degraded image quality, meaning that the electron dose must be optimized.

Imaging low-abundance particles such as bacterial flagellar motors by ECT also requires adaptations in the data-acquisition process. Data-collection parameters such as defocus, magnification, electron dose and tilt scheme can be optimized to best address the biological question. For ‘macromolecular’ resolution (∼1–5 nm) subtomogram averages, a relatively high cumulative electron dose (of between 60 and 120 e^−^ Å^−2^) and rapid data-collection settings can be chosen, attaining a balance weighted towards higher contrast at the expense of losing higher resolution details; for higher resolution reconstructions it may be necessary to reduce the dose and correspondingly collect more data. The nominal defocus must also be adjusted depending on the desired resolution. While a low defocus of around −1 or −2 µm yields higher resolution STAs, a higher defocus improves the contrast for the alignment of particularly noisy data and simplifies particle picking. Adjustment of the number and angular distribution of images in a tilt series is particularly important to decrease the data-collection time so as to acquire sufficient numbers of particles. While smaller tilt increments increase the resolution of the tomogram (Crowther *et al.*, 1970 ▸), they also extend the acquisition time and require a reduction of the electron dose per frame. Depending on the sample and the intended resolution, the tilt increment in conventional tomography typically ranges from 0.5 to 5° (Hagen *et al.*, 2017 ▸). A tilt increment of <3° increases the data-acquisition time and the required data-storage space. A further reduction in data-acquisition time can be achieved by decreasing the maximum tilt angle. Nevertheless, these optimizations lead to a data-collection time of at least 10–15 min per tomogram. To collect a sufficient amount of particles, multi-day data-collection sessions are still necessary.

### 2.3. Reconstruction   

The manual reconstruction of hundreds of tomograms with few particles is time-consuming and laborious, demanding automation. Scripts enable the pipelining of various applications (Morado *et al.*, 2016 ▸) such as fiducial tracking with *RAPTOR* (Amat *et al.*, 2008 ▸), image processing with the *IMOD* package (Kremer *et al.*, 1996 ▸) and rapid reconstruction algorithms (Agulleiro & Fernandez, 2011 ▸), facilitating the fast calculation of tomographic reconstructions to process a large quantity of tomographic data. Although medium-resolution structure determination does not always implement the correction of the perturbation by the CTF of features at different resolutions (for a more in-depth discussion, see Ferreira *et al.*, 2018 ▸), it can also significantly improve the resolution of STAs. Multiple software packages such as *Dynamo* (Castaño-Díez *et al.*, 2012 ▸; Castaño-Díez, 2017 ▸), *PEET* (Heumann *et al.*, 2011 ▸; Nicastro, 2006 ▸), *PyTOM* (Hrabe *et al.*, 2012 ▸) and *RELION* (Scheres, 2012 ▸) can then be used to obtain high-quality subtomogram averages.

## 3. Subtomogram averaging reveals considerable structural diversity in bacterial flagellar motors   

**Figure 3:**
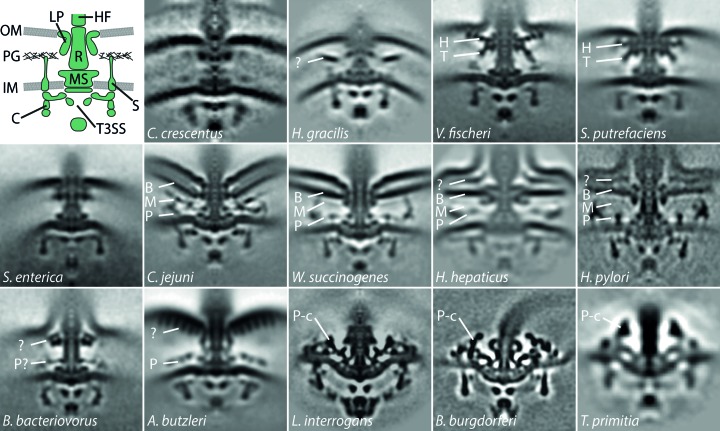
The architecture of bacterial flagellar motors reveals considerable structural diversity. Top left: schematic of the flagellar motor. Top right, middle and bottom row: micrographs show central slices (100 × 100 nm) of subtomogram averages of *C. crescentus*, *H. gracilis* (Chen *et al.*, 2011 ▸); *V. fischeri* (Beeby *et al.*, 2016); *S. putrefaciens*; *S. enterica*, *C. jejuni* (Beeby *et al.*, 2016 ▸); *W. succinogenes* (Chaban *et al.*, 2018 ▸); *H. hepaticus* (Chen *et al.*, 2011 ▸); *H. pylori* (Qin *et al.*, 2016 ▸); *B. bacteriovorus*, *A. butzleri* (Chaban *et al.*, 2018 ▸); Leptospira interrogans (Zhao *et al.*, 2014 ▸); *B. burgdorferi* (Zhao *et al.*, 2013 ▸); *T. primitia* (Murphy *et al.*, 2006 ▸). Components are labelled as follows: B, basal disk; C, C-ring; H, H-ring; HF, hook/filament; IM, inner membrane; LP, L/P-ring; M, medial disk; MS, MS-ring; OM, outer membrane; P, proximal disk; P-c, P-collar; PG, peptidoglycan layer; R, rod; S, stators; T, T-ring; T3SS, type 3 secretion system.

One of the most amenable systems to tomography, which has yielded considerable biological insights, is the bacterial flagellar motor (Fig. 3 ▸). The flagellar motor is a molecular rotary motor centred around a core cytoplasmic stator–rotor interaction that drives the rotation of a helical extracellular propeller through torque transmitted across the periplasm by an axial driveshaft (Chevance & Hughes, 2008 ▸). The stator component is a ring of inner membrane-embedded motor-protein ion channels immobilized by binding to the periplasmic peptidoglycan; ion flux drives interaction with the cytoplasmic rotor component called the C-ring. Torque applied to the C-ring is transmitted across the periplasm *via* an inner membrane-embedded MS-ring, which is connected to an axial driveshaft: the rod. To traverse the peptidoglycan layer and outer membrane, the rod passes through the P-ring and the L-ring, respectively, which act as bushings, to connect to an extracellular universal joint, called the hook, which finally transmits torque to the multimicrometre-long helical propeller: the flagellar filament. All axial structures are assembled by an integral flagellar type 3 secretion system (T3SS), with inner membrane components housed within the MS-ring together with a cytoplasmic ATPase. Until recently, much of what was known about the flagellar motor was derived from biochemical (Altegoer & Bange, 2015 ▸), genetic (Chevance & Hughes, 2008 ▸) and structural studies of purified components (Thomas *et al.*, 2006 ▸), preventing mechanistic insights into the whole, assembled molecular machine, and furthermore the majority of studies focused exclusively on the motor from the model enteric bacteria *Salmonella enterica* and *Escherichia coli*, preventing comparative insights.

Advances in ECT over the past decade have led to the observation of large, unexpected variations in flagellar motor structure (Fig. 3 ▸). The first *in situ* structure was determined in the spirochaete *Treponema primitia* from 20 motors, reaching a resolution of approximately 7 nm (Murphy *et al.*, 2006 ▸). Compared with the known flagellar structure of purified *Salmonella* motors (Thomas *et al.*, 2006 ▸), the *T. primitia* structure, and structures from related *Borrelia* species (Liu *et al.*, 2009 ▸; Kudryashev *et al.*, 2009 ▸), obtained using ECT and subtomogram averaging revealed that despite the conserved core structure, the overall architecture of the flagellar motor might be more diverse than previously expected. This was confirmed by a subsequent study comparing the flagellar motors from 11 bacterial species, revealing that in most bacteria the conserved parts of the flagellar motor resemble the *E. coli* and *Salmonella*-type motor structure, including the characteristic tripartite densities representing the rings of the export apparatus inside the cup-like structure of the cytoplasmic C-ring and the rod with P- and L-rings (Chen *et al.*, 2011 ▸), but also exhibit diverse additional structures that are discussed in more detail below.

Despite a conserved core, however, variation was also observed in the dimensions of these core components. The diameter of the C-ring varied between different species, ranging from 34 nm in *Caulobacter crescentus* to 57 nm in *T. primitia* (Fig. 3 ▸). Correspondingly, some bacteria were also observed to have distinctive stator-ring structures with variable radii and symmetries above the inner membrane aligned with the C-ring (Chen *et al.*, 2011 ▸; Murphy *et al.*, 2006 ▸). These are clearly absent in enteric bacteria but are visible in *Vibrio cholerae* (Chen *et al.*, 2011 ▸). This corresponds to results indicating that stator complexes are dynamic in enteric motors (Leake *et al.*, 2006 ▸; Fukuoka *et al.*, 2009 ▸; Baker & O’Toole, 2017 ▸), in contrast to the high-occupancy or static anchoring observed in *Campylobacter* and *Vibrio*. However, the bio­logical significance of the variations in the presence of a stator density, its symmetry and radius, and the corresponding variations in C-ring size remain unknown at the time of this study.

Strikingly, additional disk-like densities were found in the periplasmic regions of many flagellar motors: larger ones in ∊-proteobacteria such as *Campylobacter jejuni* and *Helicobacter* sp. and smaller ones in *Shewanella putrefaciens*, *Vibrio* sp., *Hylemonella gracilis* (Chen *et al.*, 2011 ▸) and *Bdellovibrio bacteriovorus* (Chaban *et al.*, 2018 ▸) (Fig. 3 ▸). The motors of the periplasmic flagellated spirochetes *T. primitia* and *Borrelia burgdorferi* discussed above exhibit large outward-facing collar structures in the cytoplasm above the inner membrane (Liu *et al.*, 2009 ▸; Kudryashev *et al.*, 2009 ▸; Murphy *et al.*, 2006 ▸; Fig. 3 ▸). At the time, the role of these additional structures composed of unidentified accessory proteins was also unclear.

## 4. A central assay: deletion mutants to understand motor architecture   

Although these advances in *in vivo* structure determination allowed the determination of the architecture of intact flagellar motors, the specific locations of proteins remained inferences from previous knowledge, leaving it difficult to decipher the locations of proteins within the *in situ* architecture.

**Figure 4:**
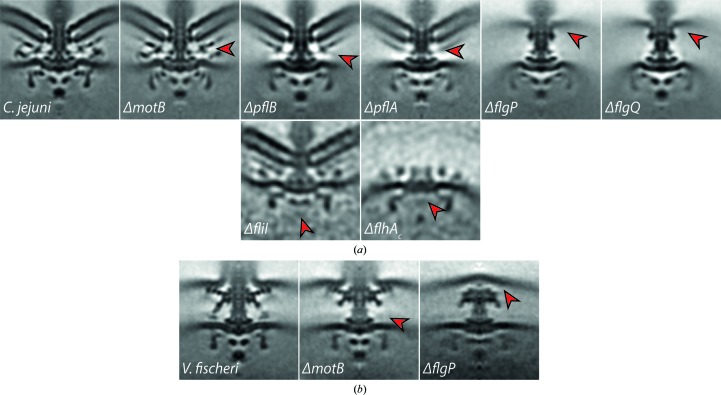
ECT and STA of deletion mutants helps to locate individual proteins within the overall motor architecture. Micrographs show central slices (100 × 100 nm) of subtomogram averages of *C. jejuni* (*a*) and *V. fischeri* (*b*) wild type and deletion mutants. Arrows point at the putative location of the respective, deleted protein that can be determined by comparison with other mutants and established biochemical data (Beeby *et al.*, 2016 ▸; Abrusci *et al.*, 2013 ▸; Chen *et al.*, 2011 ▸).

One approach to locate a protein in a tomogram is to generate an in-frame deletion of the corresponding gene and reimage the flagellar motor of the deletion mutant. The resulting subtomogram average can then be compared with the wild-type structure of the motor and examined for loss of density that may indicate the location of the protein in question (Figs. 4 ▸ *a* and 4 ▸ *b*). This method was first used to identify the location of a cytoplasmic ATPase component of the T3SS which is responsible for flagellar assembly, FliI (Fan & Macnab, 1996 ▸; Chen *et al.*, 2011 ▸). The structure of a *C. jejuni* mutant strain lacking *fliI*, which nevertheless produced sufficient motors for subtomogram averaging, lacked the lowest, cytoplasmic density of the T3SS of the *C. jejuni* wild-type structure (Fig. 4 ▸ *a*). Combined with previous knowledge about the structure of the T3SS, this indicated a putative location of FliI at the base of the flagellar motor (Chen *et al.*, 2011 ▸).

This technique has subsequently been used in many studies to locate individual protein components within flagellar motors. The next protein to be located was the integral membrane protein FlhA, a core component of the flagellar T3SS with a large C-terminal cytoplasmic domain. In *C. jejuni*, truncation of *flhA_C_* caused a cytoplasmic ring structure above FliI to disappear (Fig. 4 ▸ *a*), indicating that FlhA forms a toroidal component that mediates the interaction of the FliI–ATPase complex with the transmembrane T3SS. Indeed, correspondingly, this ring density fitted the nonameric ring of an X-ray structure of an FlhA orthologue from the *Shigella flexneri* injectisome (Abrusci *et al.*, 2013 ▸). Building on these results, multiple deletion mutants from *B. burgdorferi* were used to reveal the molecular architecture and sequential assembly process of flagellar motors (Zhao *et al.*, 2013 ▸). The location of stator-associated accessory proteins, such as FliL in *B. burgdorferi* (Motaleb *et al.*, 2011 ▸), MotAB and MotXY in *Vibrio* sp. (Beeby *et al.*, 2016 ▸; Zhu *et al.*, 2017 ▸; Fig. 4 ▸ *b*) and MotAB and PflAB in *C. jejuni* (Beeby *et al.*, 2016 ▸; Fig. 4 ▸ *a*), have also confirmed the use of this technique, as discussed further below.

## 5. Structural diversity provides a clear selective benefit: higher torque   

The ability to locate specific proteins in a subtomogram average allowed a deeper investigation of the function of additional structures in diverse flagellar motors composed of accessory proteins. Towards understanding motor evolution, a recent study probed the selective benefits of motor diversity, finding that additional motor structures serve as a scaffold to assemble larger motors that output higher torque. Torque is a measurement of rotary force, and it follows that higher torques will enable propulsion through more viscous media that would otherwise immobilize motors that produce lower torque. Motor torque varies substantially between different bacteria and correlates with their swim speed and ability to propel themselves in highly viscous media such as gastrointestinal mucus. Three different bacteria that produce different torques were compared using electron cryotomography in an effort to rationalize different torque outputs: *Salmonella* with ∼1300 pN nm torque output, *Vibrio* with ∼2000 pN nm and *C. jejuni* with ∼3600 pN nm. Using the selective deletion strategy, ECT of *V. fischeri* and *C. jejuni* not only verified the location of the stator ring in the motor structure but also enabled determination of the number of stator complexes in the stator ring. Strikingly, the number of stator complexes, and their radius from the axis of rotation, differed in these higher torque-generating species from the ∼11 stator complexes in *Salmonella* positioned ∼20 nm from the axis of rotation (Reid *et al.*, 2006 ▸; Leake *et al.*, 2006 ▸): *V. fischeri* had 13 stator complexes located at a radius of 21.5 nm from the rod, while *C. jejuni* had 17 stator complexes located 26.5 nm from the rod, and the C-rings were also correspondingly wider in both. Indeed, the number and the location of the stator complexes, combined with previously measured stator-complex force exertion, was sufficient to accurately quantitatively predict the torque outputs of structurally diverse bacteria (Beeby *et al.*, 2016 ▸).

The protein components of the additional structures have also been determined and located by deletion analysis. In *Vibrio* species FlgP has been shown to form a large ‘basal disk’ beneath, and interacting with, the outer membrane (Fig. 4 ▸ *b*). Intriguingly, in *C. jejuni* a homologous, although larger, FlgP-based basal disk also assembles under the outer membrane; a protein lattice composed of FlgQ and PflAB subsequently assembles between the basal disk and the outer membrane (Fig. 4 ▸ *a*). In both species, assembly of the wider stator ring first requires assembly of the scaffold structures, indicating that the primary role of the accessory proteins is to scaffold wider rings of additional stator complexes to exert higher torque (Beeby *et al.*, 2016 ▸).

While *Vibrio* and *C. jejuni* assemble FlgP-based stator scaffolds, parallel studies in spirochaetes suggest that high torque output has convergently evolved independently in this lineage using alternative protein building blocks other than FlgP. Spirochaete lifestyle is unusual: many are pathogens and all have flagellar filaments that coil around the cell body within the periplasm instead of passing across the outer membrane, and motor rotation is believed to drive gyration of the cell body to bore through host mucus and tissues (Charon *et al.*, 2012 ▸). Spirochaete motors are thought to output the highest torque yet discovered, rotating with a torque of ∼4000 pN nm. Spirochaete motors have a C-ring that is considerably wider than that seen in enteric motors, and 16 putative stator-complex densities are observed in a ring of corresponding width. Taken together, the location and number of stator complexes accurately predicts the measured torque of spirochaete motors (Beeby *et al.*, 2016 ▸). Whereas the FlgP-based structures form a set of stacked disks in *Vibrio* and *C. jejuni* that are responsible for wider stator-complex rings, in spirochete flagellar motors a large cup-shaped structure is seen intermediate between the rod and the stator complexes and is referred to as the P-collar (Murphy *et al.*, 2006 ▸). A comparative genomics approach identified a protein, FlbB, as a candidate component of the P-collar (Chen *et al.*, 2011 ▸), a prediction that was subsequently confirmed by deletion imaging (Moon *et al.*, 2016 ▸). As with FlgP and its associated proteins, deletion of FlbB leads to a loss of motility and failure of the stator complexes to incorporate into the motor, suggesting that spirochaetes have independently evolved a stator-complex scaffold structure to produce higher torque to facilitate their unusual lifestyle. Intriguingly, however, FlbB is only approximately 200 amino acids in length and therefore additional components are likely to be identified in the future.

## 6. Combining subtomogram averaging with phylogenetics illuminates possible evolutionary paths to higher torque   

Subsequent studies have sought to understand how these high-torque motors evolved. Naively, these motors are ‘irreducibly complex’ in that they are nonfunctional upon the deletion of individual components. Indeed, many of these components were first identified by screening for nonmotile motors resulting from mutations in genes that were not encoded in organisms with simpler motors.

A recent study revealed that the protein PflB enables the formation of the wider stator rings observed in *H. pylori* and *C. jejuni* (Chaban *et al.*, 2018 ▸). Phylogenetic analysis of bacterial species identified the descendants of intermediary ancestral states for ECT and STA imaging, resulting in visualization of the additional protein densities and their effect on the size of the stator ring in motors from different species. According to the established location of the accessory proteins in *C. jejuni*, identities could be assigned to additional densities in motor structures. Consistently, motility assays in media of different viscosities demonstrated a correlation between motor-torque output and stator-ring radius. Not only did this confirm that wider stator rings with additional stator units produce higher torque, but it also demonstrated that bacterial species lacking PflB have significantly reduced stator width and a correspondingly lower swimming ability. While *S. enterica* probably only possesses up to 11 stator units (Reid *et al.*, 2006 ▸; Leake *et al.*, 2006 ▸), *B. bacteriovorus* encodes a possible distant homologue of PflA, but not PflB, and exhibits a corresponding putative PflA disk structure that scaffolds the incorporation of 12 stator units (Chaban *et al.*, 2018 ▸). Whenever PflB is present, however, the stator ring increases to 17 ± 1 stator units: 16 stator units in *Arcobacter butzleri*, 17 in *C. jejuni* and *Wollinella succinogenes*, and 18 in *H. pylori* (Chaban *et al.*, 2018 ▸).

**Figure 5:**
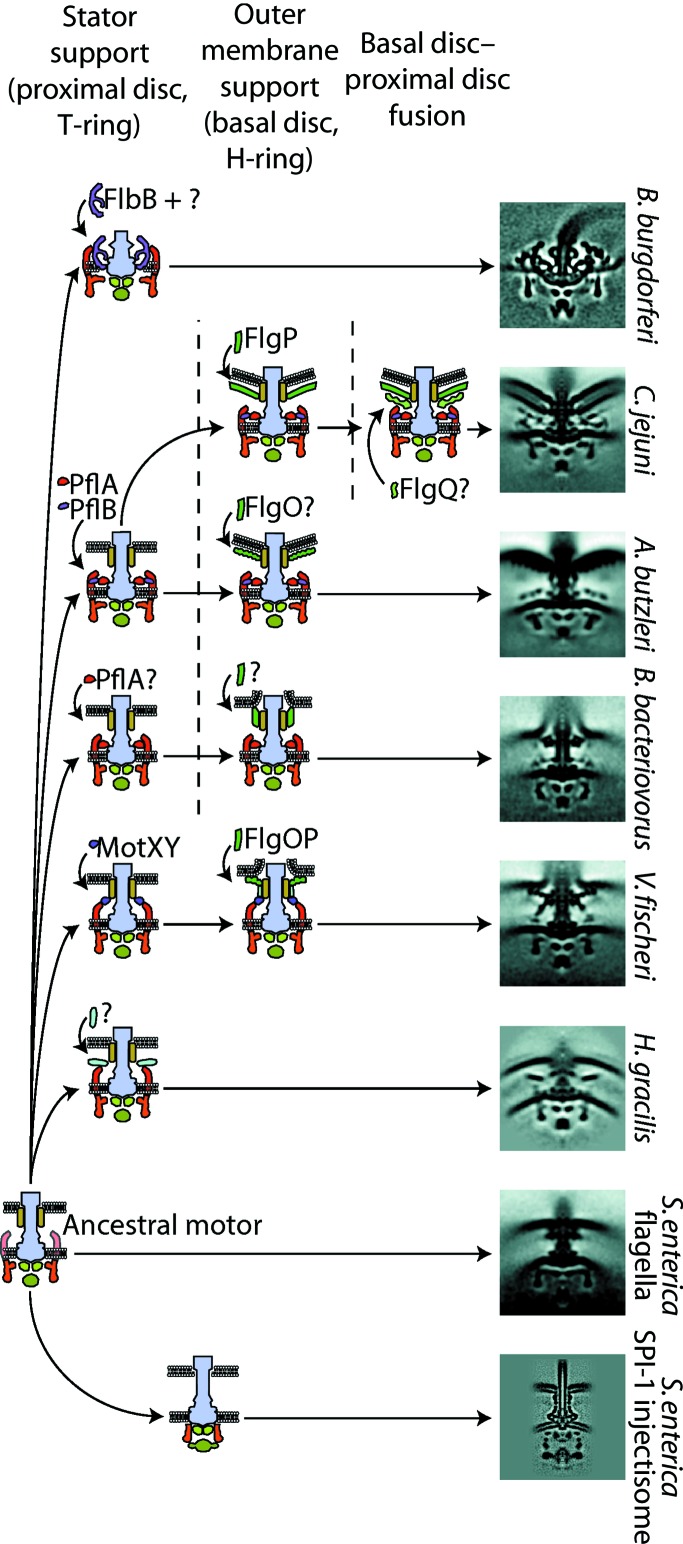
Proposed model for the evolution of the bacterial flagellar motor inferred from ECT and STA data. ECT and STA have indicated multiple pathways for the acquisition of additional accessory proteins resulting in improved stator support, increased outer membrane support and finally, in the case of *C. jejuni*, a basal disk–proximal disk fusion. ECT has also revealed structural and evolutionary insights into degenerate flagellar motors that have become injectisomes: virulence-factor delivery systems that are used by many pathogenic bacteria. Ancestral states have been inferred from representative subtomogram averages on the right coupled with phylogenetic studies (Chaban *et al.*, 2018 ▸; Beeby *et al.*, 2016 ▸).

These data also suggest a possible evolutionary pathway for the acquisition of the accessory proteins seen in *C. jejuni*-type motors (Fig. 5 ▸). Assuming an ancient flagellar motor with a relatively simple motor structure, as found in *E. coli* or *S. enterica*, the first step involves the emergence of a periplasmic disk around the rod just above the inner membrane which scaffolds and stabilizes the stator ring. Indeed, this has occurred independently at least three times and includes MotXY forming the T-ring in *Vibrio* sp., unknown proteins in *H. gracilis* and PflB in ∊-proteobacteria. In a second step, an outer membrane-associated basal disk consisting of the protein FlgP was recruited; the exact role of this disk is unclear but may be to act as an additional support anchored to the outer membrane. In a third step these two disk structures fuse to form the contemporary wider stator-complex scaffold. This fusion step was effectively a functional sidestep for both previously independent rings, which became mutually co-dependent, *i.e.* irreducibly complex (Chaban *et al.*, 2018 ▸).

## 7. Recent advances push resolution and reveal insights into the evolution of injectisomes as degenerate flagellar motors   

Another intriguing aspect of flagellar evolution that ECT has provided insights into is the degeneration of an ancestral motor to form the hypodermic syringe-esque ‘injectisome’ complex used by many pathogens. Injectisomes, also referred to as type III secretion systems, are used by diverse pathogens to inject virulence factors into host cells to hijack their physiology. Phylogenetic studies indicate that injectisomes are degenerate flagella that have lost their stator complexes and have adapted their flagellar filament to become a short, rigid, hollow needle for virulence-factor delivery (Abby & Rocha, 2012 ▸; Fig. 5 ▸). ECT studies of injectisomes requires considerable sample optimization, as many pathogens (for example *Salmonella*, *E. coli* and *Yersinia* species) are too thick for high-resolution imaging. Successful studies have employed minicell systems (Hu *et al.*, 2015 ▸; Kawamoto *et al.*, 2013 ▸) or selected thin bacteria that are more amenable to imaging (Nans *et al.*, 2015 ▸).

One of the most significant contributions of ECT to understanding injectisome function and evolution has been the visualization of a remnant of the C-ring that is still critical for injectisome function (Hu *et al.*, 2015 ▸, 2017 ▸). This insight required high-resolution technical tour-de-force studies, necessitating the collection of an order of magnitude more data than most previous studies. In the highest resolution study to date, the structure of the *Salmonella* SPI-1 injectisome was determined to 17 Å resolution (Hu *et al.*, 2017 ▸). To achieve this result, genetic techniques were used to produce *Salmonella* minicells with increased numbers of injectisomes, and tilt series were acquired using dose fractionation, motion correction and automated reconstruction of CTF-corrected data. The unprecedented 17 Å resolution final average was composed of thousands of subtomograms and provided high-resolution images of the composition of the vestigial C-ring. This and other studies demonstrate that this vestigial C-ring no longer forms a ring but rather a ring of six ‘pods’. The flagellar C-rings function to anchor the FliI ATPase complex and sort export substrates in addition to rotation and directional switching, and it is clear that the injectisome has retained a vestigial C-ring to retain these functions that are essential for assembly and virulence-factor secretion.

## 8. Future prospects   

Future prospects for understanding bacterial flagellar motors are significant as the capabilities of ECT continue to mature. The deliverables from ECT are fairly straightforward: better data, and more of it. The impacts of these deliverables will be major advances in understanding flagellar assembly, mechanism and evolution.

The most immediate contributor to higher resolution subtomogram averages will be higher quality tilt-series images, producing higher resolution tomograms and in turn producing higher resolution subtomogram averages. The introduction of improved direct electron detector cameras will be the most significant aspect of higher resolution images. The combination of robust phase plates and energy filters will further improve resolution by boosting the signal-to-noise ratio and the contrast in tomograms, enabling image acquisition closer to focus yet with high contrast (Fukuda *et al.*, 2015 ▸). This would allow the collection of higher resolution tilt series with lower electron doses, resulting in reduced electron damage. Nevertheless, recent algorithms to compensate for electron-induced specimen warping promise to mitigate for some aspects of electron damage (Fernandez *et al.*, 2018 ▸). Furthermore, three-dimensional CTF correction will fully compensate for resolution attenuation resulting from ignoring defocus modulation as a function of sample depth (Turoňová *et al.*, 2017 ▸). Finally, a recently developed, improved tilt scheme provides better data (Hagen *et al.*, 2017 ▸) which may be further optimized.

Faster data acquisition will synergize with higher quality images to produce higher resolution subtomogram averages. Higher frame-rate direct electron detectors will not only provide better motion correction and faster image acquisition, but also enable the development of stable tilt stages that are capable of collecting tilt series in seconds not minutes. Such an increase in throughput will enable the routine collection of thousands of cryotomograms and will pave the way for routine subnanometre structural determination. Such rapid data collection will also require reliable sample preparation (for example the SpotItOn approach; Jain *et al.*, 2012 ▸), the ability to seamlessly switch between grids during a data-collection session, and algorithms for automated target selection.

Even with the advances described above, it may not always be possible to accurately build a pseudo-atomic model into subtomogram average structures, and a range of hybrid methods will need further development. Deletion analysis has been invaluable to recent studies, but is inherently limited in its capability to positively identify a structure; the development of a robust tagging system to rationally insert additional domains for positive identification will be important. Furthermore, coevolutionary approaches to identify protein binding surfaces will reduce the ambiguity in modelling binding interfaces.

These developments promise to facilitate significant insights. Given the ability to acquire *in situ* structures to subnanometre resolution, the gap between structural and cellular biology will be bridged, enabling the construction of complete pseudo-atomic models of flagellar motors to understand their molecular mechanisms. As seen with the resolution revolution in single-particle analysis, the ramifications of faster data collection will be considerable and go beyond simply reducing the time required to collect a data set, rendering previously intractable questions about flagellar mechanism and evolution possible.
